# Unraveling the Landscape of Pediatric Glioblastoma Biomarkers: A Comprehensive Review of Enhancing Diagnostics and Therapeutic Insights

**DOI:** 10.7759/cureus.57272

**Published:** 2024-03-30

**Authors:** Vedant R Katole, Meghali Kaple

**Affiliations:** 1 Department of Biochemistry, Jawaharlal Nehru Medical College, Datta Meghe Institute of Higher Education and Research, Wardha, IND

**Keywords:** imaging biomarkers, non invasive biomarkers, epigenetic alterations, multi omics integration, tp53 mutations, idh mutations, pediatric glioblastoma, tumor biomarkers

## Abstract

Glioblastoma, the most common and aggressive form of primary brain tumor, poses significant challenges to patients, caregivers, and clinicians alike. Pediatric glioblastoma is a rare and aggressive brain tumor that presents unique challenges in treatment. It differs from its adult counterpart in terms of genetic and molecular characteristics. Its incidence is relatively low, but the prognosis remains grim due to its aggressive behavior. Diagnosis relies on imaging techniques and histopathological analysis. The rarity of the disease underscores the need for effective treatment strategies. In recent years, the quest to understand and manage pediatric glioblastoma has seen a significant shift towards unraveling the intricate landscape of biomarkers. Surgery remains a cornerstone of glioblastoma management, aiming to resect as much of the tumor as possible. Glioblastoma’s infiltrative nature presents challenges in achieving a complete surgical resection. This comprehensive review delves into the realm of pediatric glioblastoma biomarkers, shedding light on their potential to not only revolutionize diagnostics but also shape therapeutic strategies. From personalized treatment selection to the development of targeted therapies, the potential impact of these biomarkers on clinical outcomes is undeniable. Moreover, this review underscores the substantial implications of biomarker-driven approaches for therapeutic interventions. All advancements in targeted therapies and immunotherapy hold promise for the treatment of pediatric glioblastoma. The genetic profiling of tumors allows for personalized approaches, potentially improving treatment efficacy. The ethical dilemmas surrounding pediatric cancer treatment, particularly balancing potential benefits with risks, are complex. Ongoing clinical trials and preclinical research suggest exciting avenues for future interventions.

## Introduction and background

Pediatric glioblastoma poses a formidable challenge in the field of oncology due to its aggressive nature and limited treatment options. When compared to normal brain tissue, the perfusion, blood volume, and penetration of the high-grade glioma vasculature are all higher. The emergence of biomarkers as potent tools for early detection, accurate classification, and prognosis assessment has ignited new hope in the battle against this devastating disease. This review embarks on an in-depth exploration of the intricate world of pediatric glioblastoma biomarkers, highlighting their potential to reshape diagnostics and revolutionize therapeutic strategies. Gliomas are the most common CNS tumors, accounting for about 80% of all malignant tumors and 30% of all primary brain ones [1]. Biomarkers, defined as measurable indicators of biological processes, offer an avenue to unlock the mysteries of pediatric glioblastoma. These biomarkers, spanning genetic, molecular, proteomic, and imaging domains, hold immense promise in addressing diagnostic challenges and providing insights for tailored treatment plans. Biomarkers, ranging from genetic mutations to imaging signatures, unveil hidden facets of the disease, offering glimpses into its biology, behavior, and potential vulnerabilities. Their emergence as critical tools for early detection, precise classification, and prognostic stratification promises to shift the landscape of pediatric glioblastoma management from a one-size-fits-all approach to a personalized, targeted approach.

The significance of these biomarkers lies not only in their ability to refine clinical decision-making but also in their potential to lead a change in personalized medicine. The poor five-year overall survival rate for children with pediatric high-grade glioblastoma (HGG), which includes diffuse midline glioma (DMG) and glioblastoma multiforme (GBM), is only 20% [2]. Genetic mutations, such as isocitrate dehydrogenase (IDH) mutations and tumor protein p53 (TP53) alterations, have emerged as crucial biomarkers in pediatric glioblastoma. The WHO classification includes IDH, indicating that it has attracted a lot of attention since its discovery in human gliomas. Around 80% of grade II and III astrocytomas, oligodendrogliomas, secondary GBM, and tumors that develop from lower-grade gliomas exhibit alterations in the genes responsible for IDH1 or, less frequently, IDH2 [3]. IDH1 and IDH2 are enzymes that rely on NADP, and in high-throughput sequencing of GBM, a new IDH1 mutation was found. It causes the substitution of arginine with histidine at position 132 of the protein (R132H) at gene position 395 (G395A) [4]. Lower-grade brain tumors originating from astrocytes and oligodendrocytes include IDH-alternate codeleted oligodendroglioma, IDH-mutant intact astrocytoma, and IDH-wild-type astrocytoma [3]. Molecular characterization is crucial in diagnosing these malignancies because it provides a better threat classification than tumor grade alone [5]. TP53 mutations are comparatively less common in juvenile glioblastoma cases than in adult ones, but when they do occur, they can have a major impact on the course of the illness. Therapeutic approaches to replicate or restore TP53's typical function are being investigated. These may involve gene therapies, small-molecule compounds, or immunotherapeutic approaches. The nature and consequences of TP53 mutations vary. Comprehending these modifications is imperative in order to unravel the fundamental processes propelling tumor development within the pediatric demographic. 

Based on patterns of messenger RNA (mRNA) expression, detailed genomic studies have discovered several molecular subtypes of juvenile glioblastoma. Potential mRNA biomarkers for subtype categorization and prognosis are commonly identified by integrated analysis, which also identifies specific genes linked to these subtypes. The diagnostic and prognostic use of particular microRNA (miRNA) signatures in juvenile glioblastoma is being investigated. The irregularities of miRNAs present a therapeutic opportunity in addition to diagnostic information. miRNA-based treatments provide a new direction for precision medicine in the treatment of pediatric glioblastoma by attempting to modify particular targets implicated in the development and spread of tumors. Liquid biopsies are becoming more popular as non-invasive monitoring and diagnostic techniques because they can analyze circulating RNA in body fluids. The combination of data from genomes, transcriptomics, and proteomics, along with other omics platforms, improves the discovery of comprehensive RNA-based biomarker signatures. For the purpose of creating precise and individualized treatment plans catered to the unique molecular profile of every juvenile glioblastoma patient, this multi-omics approach is essential. The potential to revolutionize our approach to this challenging condition underpins the exploration of biomarkers for pediatric glioblastoma. The significance of the molecular markers lies not only in their ability to refine clinical judgment but also in their ability to serve as personalized guides in an era of precision medicine. No further medications have been added to the standard of care (SOC) for GBM to date, which involves maximum surgery for resection, followed by radiation and temozolomide chemotherapy. In randomized clinical trials, targeted medications and antiangiogenic therapy have not been able to demonstrate survival advantages [6]. It is clear that pediatric glioblastoma, an enigmatic as well as violent central nervous system cancer, is an intricate puzzle that challenges clinicians, researchers, and families alike. The urgency to unravel its molecular intricacies and devise innovative strategies is paramount due to its formidable nature and limited treatment options. Genetic mutations, pivotal biomarkers in pediatric glioblastoma, offer key insights into the underlying mechanisms driving the disease. Various subcategories of biomarkers have been identified based on their potential uses.

## Review

Search methodology

**Figure 1:**
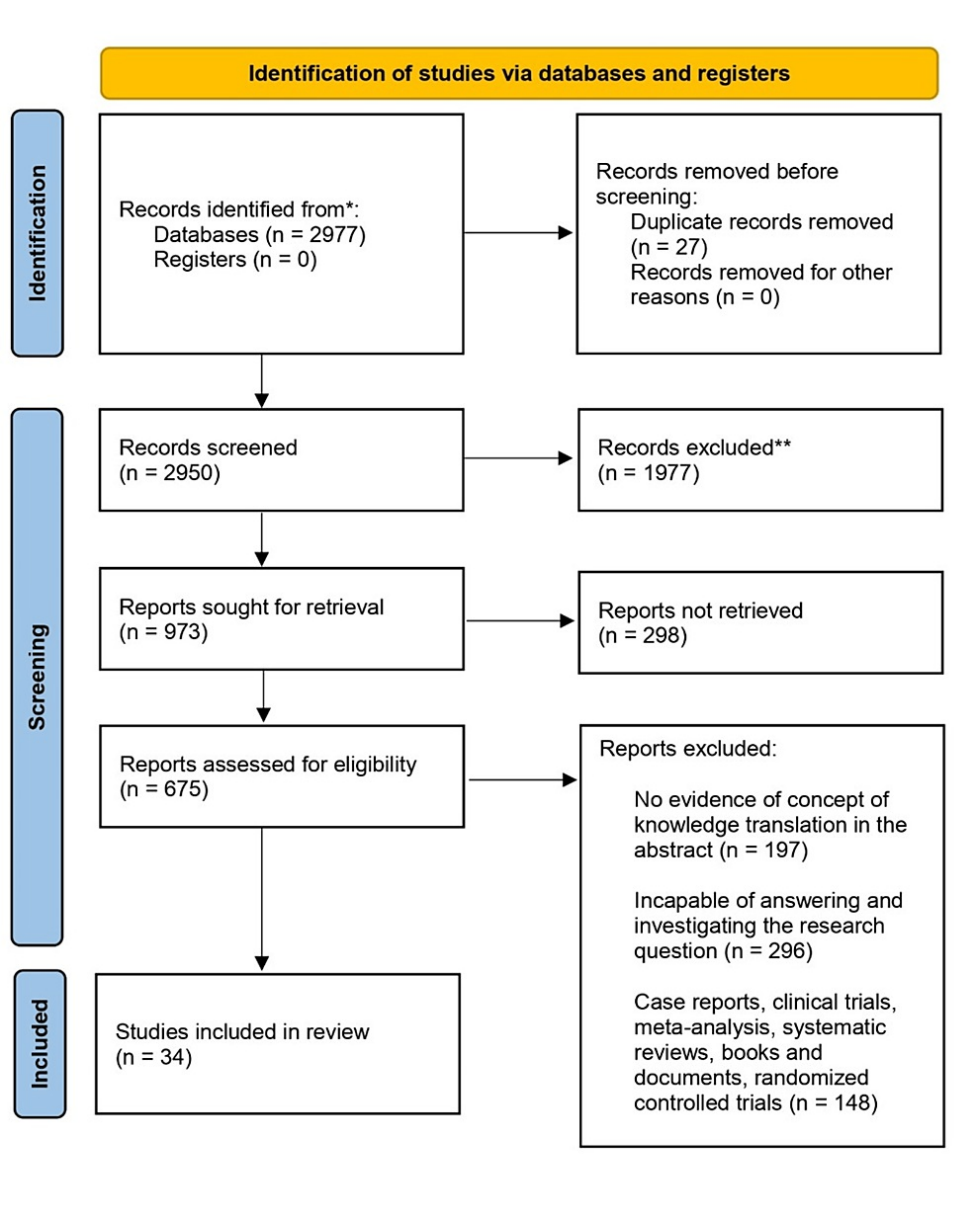
The selection process of articles used in this study Adopted from the Preferred Reporting Items for Systematic Reviews and Meta-Analysis (PRISMA)

We conducted the review through PubMed and Google Scholar in July 2023 using keywords such as “pediatric glioblastoma”, “tumor biomarkers”, “multi-omics integration”, “tp53 mutations”, “IDH mutations”, “epigenetic alterations”, “non-invasive biomarkers”, and “imaging biomarkers” for articles on pediatric glioblastoma biomarkers. In addition, we searched through the bibliographies of all relevant papers for key references. Before reading the full texts, the reviewer examined the titles as well as the abstracts of the extracted publications against the inclusion and exclusion criteria. Published English-language papers were taken into consideration for inclusion. A comprehensive literature search strategy was devised to find relevant information about the mentioned topic. The details about the search methodology have been mentioned in a Preferred Reporting Items for Systematic Reviews and Meta-Analyses (PRISMA) flowchart in Figure 1.

Molecular biomarkers in pediatric glioblastoma

For each of the several biomarkers utilized in pediatric glioblastoma, there is some kind of evidence-quality critique. The current evidence is susceptible to several limitations due to the high potential for partiality in the latest research and uncertainties regarding its relevance, particularly in assessing reaction to treatment status based on reference norms for illnesses at re-operation. In juvenile glioblastoma, molecular biomarkers are important markers acquired from the molecular features of the tumor. Liquid biopsies, immunological markers, metabolic indicators, transcriptomic signatures, miRNA and non-coding RNA signatures, epigenetic modifications, immune markers, and integrated techniques are a few of these. Although a number of molecular markers are being suggested as possible GBM biomarkers, marker heterogeneity has slowed and hindered their adoption in clinical settings. The precise role of molecular biomarkers in glioma resection has not been well studied. Since different glioma forms vary in their aggressiveness, creating more prognostic indicators may help with patient care. Traditional imaging techniques, which typically rely on the experience of the analyst, are made much more effective when combined with molecular markers such as MGMT gene promoter methylation, IDH1 and IDH2 gene mutations, and 1p/19q codeletion. Comprehending the many molecular indicators associated with juvenile glioblastoma is crucial in order to customize treatment approaches to the distinct features of each patient’s tumor. When making clinical decisions, evidence-based medicine mandates that the most potent external scientific proof be used. For instance, patients with newly diagnosed GBM who received bevacizumab along with conventional therapy did not show any survival advantage. Although biomarkers may meet several needs for multiple applications, it is imperative to collect data to support their usage. Research is still being done to enhance our understanding and find novel molecular targets that may help improve the prognosis for children with glioblastoma.

*IDH Mutations*

Isocitrate dehydrogenase, or IDH, is an essential enzyme for cellular metabolism. Changes in the DNA sequence of the IDH gene result in mutations that affect the structure and functionality of the IDH enzyme. Comparing IDH mutations to adult glioblastomas, they are comparatively uncommon. It is more usual to find wild-type (non-mutated) IDH in pediatric glioblastomas. The IDH mutation is a significant molecular biomarker in glioblastoma. Along with somatic mutations in the genes encoding two of its isoforms of isocitrate dehydrogenase (IDH1 and IDH2), the IDH kinship of enzymes catalyzes the conversion of isocitate to ketoglutarate while also changing nicotinamide adenine dinucleotide phosphate (NADP+) into reduced NADP [7]. It is well recognized that mutations in IDH change cellular metabolism, affecting the balance of metabolites as well as encouraging oncogenesis. When compared to patients with IDH-wild-type tumors, patients with glioblastomas that are IDH-mutant often have longer overall survival times. Compared to individuals with IDH-wild-type tumors, people with IDH-mutant gliomas have various treatment choices, like chemotherapy and radiation, and tend to respond better to particular medicines. The prognostic significance of IDH mutations in juvenile patients is less evident. Amino acids inside IDH are the primary source of most mutations, while other alterations at these or nearby locations are also seen [8]. The evaluation of glioblastoma patients as a whole is increasingly using molecular profiling, which includes IDH status. Cancer-associated IDH1 mutations produce 2-hydroxyglutarate (2HG) rather than ketoglutarate, and this change alters cancer metabolism and causes oxidative stress [9]. A diffuse or anaplastic astrocytoma serves as the normal precursor for IDH-mutant glioblastoma or secondary glioblastoma [10]. IDH's neo-enzymatic activity can increase reactive oxygen species (ROS) levels, hypermethylate certain DNA regions, and encourage the growth and spread of tumors [11]. The rest of the diffuse and anaplastic astrocytoma, IDH-wild-type, which displayed a passive clinical course, had been low-grade gliomas, which include pilocytic astrocytoma, glioneuronal tumors, and diffuse gliomas of the pediatric type, according to more accurate molecular analyses, including DNA methylation profiling [12]. If an imaging technique can distinguish patients between responder as well as non-responder subsets, it should be integrated to serve as a neural imaging “companion diagnostic” in the clinical studies of medicines that target the IDH1 targeting drugs [13]. Regardless of progress, there are still issues in comprehending the precise function of IDH mutations in pediatric glioblastoma. Due to the low incidence of these mutations in pediatric instances, extensive cooperative efforts are necessary to gather enough data for thorough analysis. The precise function of IDH mutations in juvenile glioblastoma is still being investigated.

*TP53* *Mutations *

Tumor protein 53, or TP53, is an essential tumor suppressor gene that modifies the cell cycle, DNA repair, and programmed cell death. The TP53 gene can become mutated, changing the DNA sequence, which leads to an abnormal or non-functional p53 protein. The lack of the p53 protein's typical tumor-suppressive properties is partly caused by TP53 mutations. TP53 mutations are frequently linked to certain glioblastoma molecular subtypes. The co-occurrence of several other genetic abnormalities, including mutations in the retinoblastoma (RB) pathway, leads to the diversification of TP53-mutant glioblastomas. During carcinogenesis, common triggers for TP53 activation include DNA damage, genotoxicity, oncogene activation, abnormal growth signals, and hypoxia [14]. The effectiveness of traditional therapeutic techniques is impacted by tumors having TP53 mutations that demonstrate resistance to certain therapies. TP53, with both wild-type and mutant forms, represents a combination of normal tissue, including endothelial cells or inflammatory cells, and the tumor [15]. Targeting TP53-related pathways is being investigated by small medicines, gene treatments, and experimental methods like CRISPR/Cas9 gene editing. Many chemotherapy drugs work by causing DNA damage that activates intact p53, making TP53-mutated or absent tumor cells resistant to these drugs [16]. The tumor microenvironment, immunological responses, and general tumor behavior are all impacted by the interaction between TP53 mutations and different molecular changes. Combining many biomarkers yields a more complete molecular profile, which helps with prognostic evaluations and therapy planning. Technological developments in sequencing and molecular profiling will lead to a more comprehensive knowledge of TP53 mutations in juvenile glioblastoma. Except for the SHH-type, p53-activated subgroup, TP53 alterations are uncommon in oligodendrogliomas as well as well-circumscribed astrocytic malignant cells like pilocytic astrocytomas, polymorphic xanthoastrocytomas, and ependymomas, along with embryonal tumors like medulloblastoma [17]. There is still much to learn about the discovery of new therapeutic targets as well as the creation of creative treatment plans. In summary, the biological processes and clinical behavior of juvenile glioblastoma are significantly influenced by TP53 mutations. The field of precision medicine for pediatric glioblastoma will probably change in the future as more is learned about the functional effects of TP53 mutations and the manner in which they affect therapy.

*RNA* *and* *MicroRNA* *Biomarkers* 

RNA biomarkers are diverse RNA molecules, which include long non-coding RNA (lncRNA), mRNA, and miRNA, that function as markers of gene expression, regulation, and cellular activities in pediatric glioma cells. Miniature non-coding RNA molecules known as miRNAs are essential for post-transcriptional gene control. RNA polymerase II and promoter elements strongly influence the transcription of miRNAs, which are primary anti-sense interactors derived from primary transcripts (Pri-miRNAs) [18]. RNase 3 processes Pri-miRNA in the nucleus and then transports it to the cytoplasm to produce mature miRNAs, which are typically less than 25 nucleotides long [19]. Since lack of regulation of miRNAs is frequently seen in cancer, including glioblastoma in children, these molecules are crucial biomarkers for the identification of many diseases. mRNA biomarkers offer insights into the patterns of gene expression, which correlate with the functioning of particular pathways alongside biological activities in juvenile glioblastoma cells. This emphasizes the critical role that miRNAs play as transcriptional and post-transcriptional agents. Even though they do not encode proteins, lncRNAs have the ability to control gene expression and add to the complex molecular landscape of juvenile glioblastoma. By attaching to target mRNAs and causing translational blockage or mRNA degradation, miRNAs control the expression of certain genes. miRNAs may operate in a series of incidents resulting in oncogenesis by operating either as suppressors of tumors or as oncogenes by alternately acting as protein-coding oncogenes or even tumor suppressor genes [20]. In the mammal differential pathway, miRNAs act as switches, and protein synthesis as well as changed expression result in the progression of tumors, which includes GBM [18]. Papagiannakopoulos used pathway analysis to discover miR-21’s relationship to mechanistic function in GBM. Their research found a direct correlation between miR-21’s ability to inhibit apoptosis by modulating the activity of P53 and transforming growth factor-β (TGF-β) [21]. Because cancer is a complicated disease, the presence of specific miRNAs influences its progression. For instance, miR-21 and miR-26, which have been expressed excessively in GBM, might affect the mRNA of numerous P53-related genes [22]. Cell cycle stoppage and cell death are inhibited as a result of reduced miRNA expression. Recently, it was discovered that a cluster of miRNAs, including miR-9*, miR-23a, miR-27a, and miR-9-3p, can differentiate between the proneural and mesenchymal subtypes of primary GBM [23]. Major GBM regulation mechanisms include proliferation, division, apoptosis, migration, and invasion involving miRNAs [24]. Current research is being done on the use of circulating RNA, particularly miRNAs, in liquid biopsies (blood or cerebrospinal fluid). Real-time data on the course of an illness and its response to therapy may be obtained by non-invasive surveillance of RNA biomarkers. Researchers can examine the effects of modifying RNA biomarkers on tumor behavior using cell lines, patient-derived xenografts (PDX), and different experimental models. To sum up, RNA and miRNA biomarkers in pediatric glioblastoma provide a wealth of data for prognosis, diagnosis, and treatment selection.

*Multi-Omics* *Integration* 

The practice of integrating data from several omics layers to get a deeper understanding of the illness process is known as multi-omics integration [25]. To fully comprehend the molecular landscape of pediatric glioblastoma, data from many omics disciplines, including proteomics, metabolomics, transcriptomics, and genomes, is integrated. To obtain a comprehensive understanding of the GBM, information from several biological data layers is combined. The multi-omics traits of RNA-Seq, copy number variation (CNV), reverse phase protein arrays (RPPA), and somatic mutation were grouped using a gene [26]. Several omics studies have shown genes, proteins, and metabolites associated with certain illnesses or desirable characteristics. Multi-omics research using databases such as the Chinese Glioma Gene Atlas (CGGA), the Cancer Genome Map Research Network (TCGA), and others highlights the complicated genetic makeup of GBM. Precision medicine techniques are made easier by multi-omics integration, which allows treatment regimens to be customized based on the distinct molecular profile of individual pediatric glioblastomas. A cross-modality autoencoder (CrossAE) has been proposed by deep learning (DL)-based survival analysis to incorporate multi-omics by carrying out comparable cross-modality rebuilding [27]. Dimensionality reduction (DR) is a technique that lowers a dataset's complexity while improving downstream studies' statistical power, stabilizing predictions, and lessening the need for multiple tests. In pediatric glioblastoma, these biomarkers play diagnostic, prognostic, and predictive roles that inform clinical judgment and treatment strategy. Progress in single-cell omics techniques boosts multi-omics integration up to the single-cell level. Findings are integrated and understood in a multi-omics context by mapping single-omics analysis results onto a network. Single-block integration solutions only concatenate several data matrices into one big data matrix before using a statistical analysis technique to integrate various omics datasets. This makes it possible to identify routes that are improperly regulated at the gene, protein, and metabolite levels. The availability of extensive multi-omics databases and additional data sources enables researchers to gain a deeper understanding of the genetic and molecular features of the GBM. A deeper understanding of the dynamic alterations in the molecular landscape of juvenile glioblastoma throughout time is gained via longitudinal multi-omics investigations. The relationship between molecular traits and clinical consequences is strengthened when multi-omics data is integrated with medical data like patient outcomes and therapy responses. Although each omics layer has value on its own, the majority of multi-omics combination research in a larger picture of system biology uses transcriptomics and proteomics [28]. Data from transcriptomics and metabolomics are produced by essentially distinct analytical methodologies. The integration of data from several omics platforms presents standardization, normalization, and interpretation issues. Safeguarding patient privacy and gaining informed permission for thorough molecular profiling are crucial ethical issues, especially as the focus on large-scale multi-omics investigations grows. To summarize, the integration of multi-omics in pediatric glioblastoma is a potent method for deciphering the sophisticated molecular details of the illness.

Diagnostic biomarkers in pediatric glioblastoma

The quality of the evidence currently available to assess the diagnostic accuracy of biomarkers for glioblastoma treatment response tracking is relatively low. For example, research is still being conducted to determine the many methods for distinguishing between actual tumor progression and false progression. Concurrently, a monitoring biomarker that can accurately differentiate between pseudoprogression and genuine progression would provide comprehensive information to support the challenging decision. Although little research has been done, certain studies have demonstrated the usefulness of magnetic resonance spectroscopy (MRS) in distinguishing between pseudoprogression and tumor recurrence. A variety of genetic, imaging, and histopathological markers are used as diagnostic biomarkers in juvenile glioblastoma. These encompass protein biomarkers, magnetic resonance imaging (MRI), MRS, DNA methylation patterns, and BRAF V600E mutations. When these indicators are combined, a thorough and precise diagnosis may be made, which helps doctors create specialized treatment plans for young glioblastoma patients. A diagnostic biomarker’s context of use must be carefully considered if it is to be used in prospective research or clinical practice, as opposed to being used more broadly to further scientific notions or other general purposes. The available data indicates that the approach has evolved significantly with regard to the possibility of improved MRI methods being used for biomarker monitoring. Continuous investigation persists in honing these diagnostic methodologies and discovering new biomarkers to enhance accuracy in diagnosis and prognosis. A diagnostic biomarker aids in changing how the illness is classified. In the case of biomarkers, the lack of a historical benchmark to determine whether an illness or condition is present or absent is a prevalent issue. The main obstacles to the combining of imaging and molecular genetic characteristics include variation in imaging acquisition protocols among institutions, tumor sampling, distinct regions of interest, and issues in aligning the imaging dimension with molecular profiles. At present, efforts are being made to identify new markers that might serve as therapeutic targets and enable a more precise diagnosis. Given the benefits and drawbacks of each biomarker, a combination of biomarkers may be useful to acquire non-invasive diagnostic and prognostic data.

Imaging and Non-invasive Biomarkers

The structural and functional features of juvenile glioblastoma can be visually and statistically observed with the use of imaging biomarkers. Non-invasive biomarkers are a wide variety of quantifiable indicators that may be collected without intrusive treatments. Non-invasive diagnostic methods are critical, especially in young patients, where invasive treatments might be difficult. Imaging can't differentiate between real tumor growth and treatment-related pseudo-progression, which looks like tumor growth and may resolve naturally over time [29]. Imaging offers comprehensive anatomical details on the dimensions, position, and features of juvenile glioblastoma tumors. An MRI, or magnetic resonance imaging, as well as positron emission tomography (PET), are useful in determining and analyzing pediatric glioblastoma lesions. Knowing the tumor microenvironment is aided by methods such as perfusion MRI and functional MRI (fMRI), which offer information about blood flow, tissue perfusion, and neuronal activity. miRNAs are a few of the most promising indicators for cancer detection and circulating IncRNAs, just like miRNAs, have the ability to function as diagnostic as well as prognostic indicators in glioblastoma [30]. Radiotracer-enhanced PET scans can identify regions of elevated metabolic activity, which aids in differentiating the tumor from healthy tissue. Advanced imaging techniques can provide crucial knowledge regarding a tumor’s dimension, position, and structural characteristics. Blood biomarkers that are in circulation, such as certain proteins and nucleic acids, along with metabolites, may provide information about the existence, course, or responsiveness to therapy of a malignancy. The identification of cell-free miRNAs in bodily fluids such as serum and plasma has provided a subtle source of biomarkers for a variety of disorders, including carcinoma [31]. Liquid biopsies, which include circulating tumor DNA (ctDNA) assessment, allow for the identification of tumor-specific changes without the need for tissue samples. Prompt diagnosis enables early treatment, which may improve results. Furthermore, detecting specific biomarkers in physiological fluids that include cerebrospinal fluid (CSF) or blood provides simple alternatives for diagnosing and monitoring juvenile glioblastoma. Proteins, miRNAs, and other molecular markers corresponding to the disease may be among these biomarkers. Using imaging in addition to non-invasive biomarkers together enables a more individualized approach to care. Treatment precision is increased when medications are customized based on the unique features of the tumor as identified by non-invasive techniques. Complex imaging data analysis is increasingly being done using machine learning and artificial intelligence methods. Incorporating imaging and non-invasive biomarkers within the clinical setting is a potential step toward improving the accuracy and speed of pediatric glioblastoma diagnosis. Cancer cells, known as circulating tumor cells (CTCs), are discharged into the circulation by primary tumors, where they spread to other locations and ultimately develop into metastases. While glioma metastatic dissemination is exceedingly rare, circulating tumor cells exhibiting glioma features have been found in various investigations. DNA fragments known as circulating cell-free DNA (ccfDNA) circulate in bodily fluids in low amounts. ctDNA is the term for DNA fragments produced by necrotic or otherwise apoptotic cells in cancer patients. Numerous investigations have demonstrated the potential for identifying tumor-associated mutations in ctDNA in individuals suffering from primary CNS malignancies, such as GBM. Another class of molecules released into bodily fluids from cells and tissues are called cell-free RNAs. Their source may be a vesicle-free RNA-binding protein-dependent mechanism, active secretion via membrane-bound vesicles, or latent secretion from necrotic or apoptotic cells. Also, liquid biopsy makes it possible to collect repeatable, non-invasive samples, enabling real-time patient monitoring during therapy. Nevertheless, no clinically validated circulating biomarkers for the management of GBM have been found so far. A trustworthy substitute for identifying the histology and molecular subgroups of gliomas is the MR radiomics-based approach. Texture analysis and its broader radiomics refers to a range of image analysis methods, some of which are invisible to the human visual system, that measure variations in surface intensity or patterns. The most thorough research on cerebral gliomas has been done on brain tumors, utilizing MR-based texture analysis (MRTA) to ascertain the relationship between different clinical parameters and MRTA characteristics. Significant relationships between imaging characteristics and genetic profiles in GBM can be further uncovered by using MR radiomics. The field of "molecular imaging" and radiogenomics has emerged as a result of the quantitative radiomic data taken from improved MRI, which have emerged as promising in vivo noninvasive biomarkers to estimate tumor classes and molecular variants. Multimodal MR radiomics can accurately identify the IDH and the 1p/19q status of gliomas and distinguish GBMs from low-grade gliomas (LGGs). There is increasing evidence that MRI phenotypes may be used to investigate the underlying genotypes, which may have applications in discriminating tumor molecular profiles driven by imaging characteristics. In addition to paving the way for more precise diagnostic and prognostic evaluation, the growing use of artificial intelligence (AI)-based models will investigate the utility of higher-level cMRI radiomic features, which may more closely align with microscopic findings and serve as a substitute for cutting-edge aMRI markers in the description of tumor tissue microenvironments. The creation of multimodal biomarker panels, which combine non-invasive molecular markers with imaging characteristics, may provide a more dependable and durable method for characterizing juvenile glioblastoma. Future studies will probably concentrate on combining data from several imaging modalities along with non-invasive biomarkers to produce a thorough and dynamic profile of juvenile glioblastomas.

*Epigenetic Alterations in Pediatric Glioblastoma* 

These are inheritable variations in gene expression without changes to the fundamental DNA sequence. Gene activity is greatly influenced by epigenetic changes such as histone modifications, DNA methylation, and non-coding RNA expression. Changes in regular epigenetic patterns have a role in the onset and progression of juvenile glioblastoma. Epigenetic changes are becoming more well-recognized as important determinants in the genesis and advancement of juvenile glioblastoma. Understanding epigenetic changes in pediatric glioma provides far-reaching consequences regarding diagnosis and prognosis, as well as the emergence of targeted therapeutics. One typical epigenetic change in juvenile glioblastoma is the hypermethylation of promoter regions, which silences tumor suppressor genes. The development of tumors and unchecked cell proliferation are facilitated by this lack of tumor suppressor activity. The level of methylation in the MGMT promoter is linked to age-related brain tumors, prognosis, and immune resistance, but it's not the first biomarker found due to epigenetic changes [32]. Different clinical outcomes may be linked to the existence or lack of certain methylation patterns, which enables doctors to make well-informed prognostic judgments. Abnormal DNA methylation variations distinguish pediatric glioblastoma. The introduction of groups of methyl to certain areas of DNA, which is known as hypermethylation, can result in the silencing of tumor suppressor genes, which results in decreasing their potential to regulate cell growth and division. Changes to the histone proteins, which wrap DNA, are also involved in epigenetic changes. Histone alterations can change the structure of chromatin, making some sections of the genome accessible in some manner for gene expression. The changes in various processes of histone acetylation, along with methylation and phosphorylation, have been found in pediatric glioblastoma. The possibility of epigenetic-targeted medications, such as histone deacetylase inhibitors and DNA methyltransferase inhibitors, to treat glioblastoma is being studied. Specific histone changes can indicate the extreme nature as well as the response to medication for a malignant tumor. The methylation of histone has various consequences for pediatric as well as adult glioblastoma sufferers [33]. Focusing on these histone changes, along with epigenetic treatments, has therapeutic potential. The incorporation of epigenetic information into precision medicine approaches facilitates a more thorough comprehension of the molecular characteristics of juvenile glioblastoma. The goal of an additional study is to pinpoint certain epigenetic modifications that can be the focus of therapeutic intervention. Understanding the relationships between signaling pathways and epigenetic alterations in juvenile glioblastoma may help identify new targets for treatment.

Therapeutic insights 

Current Treatment Strategies for Pediatric Glioblastoma

Juvenile glioblastoma is a kind of high-grade brain cancer that affects children and adolescents. Surgical procedures, radiation therapy, and chemotherapy are used as treatment approaches for pediatric glioblastoma (pGBM). Owing to the tumor's aggressive character, a multidisciplinary strategy is frequently used to treat the disease's systemic and local components. Because of the lethal character of the disease and the requirement for stable and efficient treatment, along with the limiting long-term negative effects, treating juvenile glioblastoma continues to be a substantial task. Radiation therapy, which targets leftover tumor cells after surgery, is critical in the treatment of pGBM. It lessens the chance of recurrence and aids in the treatment of local illnesses. Juvenile glioblastoma has a significant level of microscopic polymorphism when viewed alongside adult GBM, but it also exists within its own subtype [34]. Resection by surgery is the most commonly used therapeutic option for pediatric glioblastoma. Brain surgeons strive to cautiously eliminate the majority of the tumor that can be removed while avoiding major injury to adjacent, normally functioning brain tissue. Patients with pediatric GBM may benefit from conformal treatment with radiation, a method that hits the tumor accurately while conserving normal brain tissue. Treatment with chemotherapy is an essential aspect of juvenile glioblastoma medication. Temozolomide is a regularly used oral chemotherapy medication. The molecular subtyping of pediatric glioblastoma is made possible by advancements in molecular profiling, which aid in the identification of certain genetic and molecular traits. Targeting tumor cells with immune system recognition and assault is a potential path for upcoming therapeutic development. Novel treatment approaches, including oncolytic viruses, gene therapies, and combination therapy, are being investigated via ongoing research. To test novel treatment modalities and improve our understanding of pGBM, clinical trial participation is essential. Pediatric diffuse gliomas are formed and maintained by obligatory genetic changes in conjunction with epigenetic modifications. Histone demethylases, histone methyltransferases, histone deacetylases (HDACs), and bromodomain inhibitors are the different epigenetic targets. Comparable HDAC and demethylase inhibitor combos have demonstrated preclinical efficacy in patient-derived xenografts, both in vitro and in vivo. Currently, a phase I study is being conducted to evaluate panobinostat as a single drug for the treatment of progressive or recurrent HGG. The proteins known as the bromodomain and extraterminal domain (BET) are examples of epigenetic readers because they attach to acetylated residues of lysine in histone tails and control biological processes such as transcriptional regulation, DNA replication, and DNA damage repair.

Other Available Options in Juvenile Glioblastoma

Immunotherapeutic techniques, including immune checkpoint inhibitors as well as chimeric antigen receptor (CAR) treatments, are being studied. These therapies use the immune system’s ability to recognize as well as combat cancer cells. Inhibitors of certain kinases or other molecules implicated in important signaling pathways are used as targeted therapy in juvenile glioblastoma patients. As the normal treatment choices have limited effectiveness, numerous pediatric glioblastoma patients and their families do get involved in clinical studies. These studies provide for the availability of novel medications and also help to expand the options for treatment choices for this uncommon and severe malignancy. In order to operate medication side effects, regulate dietary habits, and promote their well-being in general, children diagnosed with glioblastoma need extensive medical attention. Viruses known as "oncolytics" target and kill cancer cells while leaving healthy cells unharmed. Targeting cancer cells, oncolytic viruses cause them to lyse and release viral particles, which might intensify the body's defenses against the tumor. A targeted and selective therapy approach for pediatric glioblastoma is offered by oncolytic viruses, which combine immune-mediated antitumor responses with direct cytotoxic effects. To improve therapeutic efficacy and reduce resistance, several medicines with complementary modes of action are combined. Utilizing synergistic effects and lowering the likelihood of treatment resistance are the objectives. A crucial component of novel treatment approaches is enhancing the delivery of therapeutic medicines to the tumor site while reducing systemic toxicity. The goal of advances in pediatric glioblastoma medication administration is to improve the medications' capacity to penetrate the blood-brain barrier and become bioavailable in the brain. Palliative treatment becomes a priority when the tumor is incurable or all therapeutic alternatives have been explored. Palliative care experts help patients and their families cope with signs and symptoms, enhance their standard of existence, and also offer emotional assistance. Current research as well as clinical investigations are aimed at identifying novel targets for therapy as well as refining approaches to medication in order to further enhance the results regarding children and teenagers who are impacted by such a lethal tumor of the brain. Despite the difficulties, advances in treatment choices continue to provide promise regarding enhanced results in the years to come. For a thorough approach to pediatric glioblastoma diagnosis and treatment, it is essential to comprehend the complementary roles of molecular and diagnostic biomarkers. Molecular biomarkers explore the complexities of the biology of the tumor, whereas diagnostic biomarkers offer crucial data for preliminary identification, localization, and histological description. To boost potential clinical uses and improve specificity and sensitivity, more research with larger cohorts is required. A summary of all the articles included in this review is listed in Table 1.

**Table 1:** Summary of the articles included in the review NADP: nicotinamide adenine dinucleotide phosphate, IDH: isocitrate dehydrogenase, GBM: glioblastoma multiforme, WHO: World Health Organisation, IDH-TET: isocitrate dehydrogenase-ten eleven translocation, 2HG: 2-hydroxyglutarate, HGG: high grade glioma, p53: tumor protein, LFS: Li-Fraumeni syndrome, TP53: tumor suppressor gene, RNA: ribonucleic acid, MiR/miRNA/miR: micro ribonucleic acid, GDP: group lasso regularized deep learning for the survival prediction in cancer patients, IR: insulin resistance

Authors	Year	Country	Findings
Liu et al., [1]	2023	China	Adiposity, smoking, drinking alcohol and coffee consumption have no role in growth of gliomas.
Parsons et al., [2]	2009	USA	Mutations in NADP(+)-dependent isocitrate dehydrogenases coded by the IDH1 and IDH2 are seen in the majority of malignant gliomas.
Sanvito et al., [3]	2021	Italy	For forecasting molecular state, multimodality evaluation surpasses single–metric techniques.
Jones et al., [4]	2008	USA	Some of the recognized routes changed in GBMs impact a greater proportion of genes as well as patients than formerly thought.
Dehais et al., [5]	2016	France	The latest WHO histomolecular grouping of diffuse gliomas with a strong prognostic value was reported.
Bellu et al., [6]	2017	Italy	Antiangiogenic medicines did not increase survival as a whole in individuals with glioblastoma.
Chen et al., [7]	2017	USA	Gliomas are classified histologically into several molecular subgroups, each with its own nature, background and prognosis.
Yang et al., [8]	2012	China	The identification and subsequent analysis of IDH1/2 alterations in cancers resulted in the recognition of the IDH-TET pathway.
Jezek et al., [9]	2020	Czech Republic	Specific cancer metabolism is linked to 2HG enantiomers. Their production is accompanied by oxidative stress. They could be used as metabolic indication in the future.
Khasraw et al., [10]	2020	USA	A plethora of innovative medicines have shown promise efficiency in the persistent or relapsed situation to achieve more sustained responses in glioblastoma patients.
Zhang et al., [11]	2022	China	The current standard of care for newly diagnosed GBM is alkylating agent chemotherapy coupled with radiation.
Komori et al., [12]	2022	Japan	Prospective controlled trials are needed to determine the most effective treatment strategy for each patient.
Li et al., [13]	2020	China	For HGG patients, particular molecular expression cycles along with therapy responses can be visualized serially.
England et al., [14]	2013	USA	Signaling changes downstream of p53 variants as well as microRNAs are important in modifying the p53 pathway.
Cotter et al., [15]	2018	USA	Female companions of LFS patients may be at more risk of choriocarcinoma because of germline mutation in TP53 transmission from male carriers.
Merkel et al., [16]	2017	Austria	To protect the genome’s structural integrity, p53 must be restored to normal activity.
Park et al., [17]	2017	Korea	High-throughput assays that may examine numerous genes simultaneously are critical for disease diagnosis.
Lawler et al., [18]	2009	USA	MicroRNAs are involved in glioblastoma features like cell proliferation, glioma stem cell activity along with angiogenesis.
Lee et al., [19]	2003	Korea	RNase proteins like Drosha as well as Dicer play vital functions in miRNA-mediated gene control during development and proliferation processes.
Calin et al., [20]	2006	USA	Changes in miRNA genes are important in the underlying causes of many human malignancies.
Kosik et al., [21]	2008	USA	In glioblastoma cells, several oncogenes concentrate on mitochondrial apoptotic tumor suppressive genes.
Chan et al., [22]	2005	USA	MiR-21 that is abnormally expressed could lead to the cancerous traits by inhibiting the production of essential apoptosis-related genes.
Huang et al., [23]	2018	USA	Finding miRNA patterns in proximal fluids as well as exosomes is a critical step in tumor subtypes classification.
Karsy et al., [24]	2012	USA	In GBM, miRs can influence multiple processes at once, including tumor development and its attack.
Kang et al., [25]	2022	USA	A multi-omics along with non-omics analysis strategy can be used to develop complete illness risk evaluation in addition to precision medicine.
Xie et al., [26]	2019	USA	GDP may soon be useful for clinical diagnostics along with genomics-guided prognosis.
Tong et al., [27]	2020	USA	Emerging multi-omics integration frameworks can help with individualized breast cancer diagnosis along with medication.
Lee et al., [28]	2016	Sweden	Mannose is amongst the most effective metabolites in plasma for explaining variation in obesity-independent IR.
Chua et al., [29]	2019	Australia	The liquid biopsy method has a lot of promise for treating patients with GBM.
Nagy et al., [30]	2020	Hungary	The advantage of employing novel molecular markers versus standard markers is that sampling is less invasive.
Zanganeh et al., [31]	2023	USA	The utilization of biomarkers in bodily fluids shows hope in glioblastoma detection, diagnosis, as well as surveillance.
Verdugo et al., [32]	2022	Spain	To develop successful therapeutics for GBM, numerous conditions like metabolic variability, tumor spread potential, poor pharmacokinetics should be tackled.
Romani et al., [33]	2018	Italy	Epigenetic molecules have the ability to affect the adaptability of the tumor setting in glioma.
Wyss et al., [34]	2022	Switzerland	Specific as well as immunotherapeutic treatments should be included in innovative therapy regimens, perhaps leading to a better outcome in juvenile glioblastoma.

## Conclusions

Juvenile glioblastoma patients suffer through many changes. There has been a great deal of research done on the state of juvenile glioblastoma biomarkers, which has shed light on the elaborate molecular mechanisms driving this complicated illness. Patients with pediatric glioblastoma continue to have a low overall survival rate and few treatment choices, despite significant advancements in our knowledge of the disease's pathophysiology. Currently, the primary methods for diagnosing glioblastoma are imaging and histo-molecular analysis of tissue samples. Numerous miRNAs that are essential for oncogenesis, development, and invasion have been discovered to be dysregulated in GBM tissues. On the other hand, the benefit of imaging biomarkers is their objectivity; while they are seldom helpful in glioma classification, they are critical to its diagnosis. Personalized medicine is embodied by the clinical consequences of discovered biomarkers, which go beyond prognosis to include customized therapy approaches. It will be necessary to employ novel approaches and combinations that work in additive or synergistic ways, such as immunotherapies, in addition to traditional radiation and chemotherapy. More research on the genetic, protein, and metabolic alterations that take place during the development of glioma and glioblastoma multiforme is required to enhance early detection techniques and provide innovative, individually tailored anti-cancer treatments. Examining the intricate molecular pathways of GBM as a whole is necessary in the hunt for effective biomarkers of the illness.
